# Inositol Hexaphosphate and Inositol Inhibit Colorectal Cancer Metastasis to the Liver in BALB/c Mice

**DOI:** 10.3390/nu8050286

**Published:** 2016-05-12

**Authors:** Min Fu, Yang Song, Zhaoxia Wen, Xingyi Lu, Lianhua Cui

**Affiliations:** 1 Department of Public Health, Qingdao University Medical College, Qingdao 266021, China; bxfumin@163.com (M.F.); qdlhcui@163.com (L.C.); 2 School of Nursing, Qingdao University Medical College, Qingdao 266021, China; qdwenzhaoxia@126.com; 3 Basic Medical College, Qingdao University, Qingdao 266021, China; qdluxingyi@126.com

**Keywords:** inositol hexaphosphate, inositol, metastasis, ECM, angiogenesis

## Abstract

Inositol hexaphosphate (IP6) and inositol (Ins), naturally occurring carbohydrates present in most mammals and plants, inhibit the growth of numerous cancers both *in vitro* and *in vivo*. In this study, we first examined the anti-metastatic effects of IP6 and Ins using a liver metastasis model of colorectal cancer (CRC) in BALB/c mice. CT-26 cells were injected into the splenic capsule of 48 BALB/c mice. The mice were then randomly divided into four groups: IP6, Ins, IP6 + Ins and normal saline control (*n* = 12 per group). IP6 and/or Ins (80 mg/kg each, 0.2 mL/day) were injected into the gastrointestinal tracts of the mice on the second day after surgery. All mice were sacrificed after 20 days, and the tumor inhibition rates were determined. The results demonstrated that the tumor weights of liver metastases and the tumor inhibition rates were reduced in the experimental groups compared to the control group and that treatment with the combination of IP6 and Ins resulted in greater inhibition of tumor growth than treatment with either compound alone. These findings suggest that IP6 and Ins prevent the development and metastatic progression of colorectal cancer to the liver in mice by altering expression of the extracellular matrix proteins collagen IV, fibronectin and laminin; the adhesion factor receptor integrin-β1; the proteolytic enzyme matrix metalloproteinase 9; and the angiogenic factors vascular endothelial growth factor, basic fibroblast growth factor, and transforming growth factor beta in the tumor metastasis microenvironment. In conclusion, IP6 and Ins inhibited the development and metastatic progression of colorectal cancer to the liver in BALB/c mice, and the effect of their combined application was significantly greater than the effect of either compound alone. This evidence supports further testing of the combined application of IP6 and Ins for the prevention of colorectal cancer metastasis to the liver in clinical studies.

## 1. Introduction

The latest U.S. cancer statistics have revealed that colorectal cancer (CRC) is the third most common malignant neoplasm, and its incidence has increased in China in recent years because of changes in lifestyle and dietary habits [1]. The liver is the most common site of distant metastasis of CRC; liver metastasis affects CRC patient prognosis and is the main cause of death of these patients [2]. Surgical resection is the only potentially curative option for locally recurrent disease or metastatic disease localized to the liver or lungs, and the five-year overall survival rate is low (10%–25%). However, most CRC patients with liver metastasis are not eligible for surgery [3]. Therefore, early prevention of CRC metastasis to the liver is very important. The currently available metastatic CRC (mCRC) drugs cause side effects, as they do not discriminate between normal and tumor cells and therefore induce cell death in all actively proliferating cells. In contrast, inositol hexaphosphate (IP6) specifically attacks tumor cells and spares normal cells [4].

IP6 is a natural organic phosphorus compound that is present in almost all plant and mammalian cells, along with its lower phosphorylated forms with fewer phosphate groups (IP1-5). The richest sources of IP6 are wheat bran and flaxseed (0.4%–6.4%) [5]. IP6 has attracted a large amount of attention in recent years in domestic and international medical fields, and many studies have demonstrated that it has inhibitory effects on many tumor types [6]. IP6 has been found to be a potent inhibitor of experimental hepatoma. It also inhibits the growth of HepG2 cells and induces their differentiation and development into a less aggressive phenotype [7,8]. A single administration of IP6 to HepG2 cells *in vitro* has been shown to result in complete loss of the tumor-forming ability of these cells when inoculated subcutaneously in nude mice [9]. Additionally, regression of pre-existing liver cancer has been demonstrated following direct treatment with IP6. Lee *et al.* have shown that dietary administration of IP6 and inositol (Ins) significantly inhibits chemically induced rat hepatocarcinogenesis [10]. Ins is a saturated, circular, polyhydric alcohol that serves as the backbone and precursor of inositol phosphates. It is primarily used to treat diabetes, hepatitis, polycystic ovary syndrome [11,12,13], cardiovascular disease and other diseases. It exhibits moderate anticancer activity, and it synergistically enhances the inhibitory effects of IP6 on the growth of colon and mammary cancers [14,15].

Few reports on the effects of the combination of IP6 and Ins on tumor metastasis are available in the literature. Vucenik *et al.* subcutaneously inoculated mouse fibrosarcoma FSA-1 cells in mice and then administered intraperitoneal injections of IP6 (80 mg/kg) and Ins (80 mg/kg). They found that IP6 and Ins reduced the growth of the subcutaneously transplanted fibrosarcomas (FSA-1 cells) in the mice, prolonging their survival and subsequently reducing the number of pulmonary metastases [16]. Furthermore, preliminary clinical studies have demonstrated that IP6 + Ins administration in combination with chemotherapy reduces the side effects of chemotherapy and improves the quality of life of patients with breast cancer or CRC with liver metastasis [17,18]. However, the mechanism of action and effects of the combination of IP6 and Ins on tumor metastasis *in vivo* are not clear.

The tumor metastatic process includes migration across the basement membrane, intravasation into vessels, transport in circulation, adhesion to endothelial cells, extravasation through vessel walls, colonization, and proliferation in response to organ-specific factors at new sites. In the present study, we first evaluated whether IP6 and Ins inhibited tumor growth in a mouse metastatic tumor model. We also attempted to identify whether these compounds influence the development of liver metastasis from CRC by regulating the expression of related factors over the course of tumor metastasis. Moreover, the mechanisms underlying the inhibitory effects of IP6 and Ins on liver metastasis were explored.

## 2. Materials and Methods

### 2.1. Reagents

IP6 (MB7073) was purchased from Dalian Meilun Biotech Co., Ltd. (Dalian, China). Ins (B20581) was purchased from Shanghai Yuanye Bio-Technology Co., Ltd. (Shanghai, China). The following antibodies were used for immunohistochemical analyses: rabbit anti-transforming growth factor beta (TGF-β) antibody (bs-0486R), rabbit anti-integrin beta 1 antibody (Beijing Bioss Bio-Technology Co., Ltd., Beijing, China), rabbit anti-human vascular endothelial growth factor (VEGF) polyclonal antibody (RAB-0157), rabbit anti-human basic fibroblast growth factor (bFGF) polyclonal antibody (RAB-0305) and mouse anti-human matrix metalloproteinase (MMP)-9 monoclonal antibody (MAB-0245) (Fuzhou Maixin Biotech. Co., Ltd., Fuzhou, China). The following antibodies were used for Western blot analyses: rabbit monoclonal anti-fibronectin (FN) (NBP1-91258) and anti-laminin (LN) antibodies (NB300-144) (Cell Signaling Technology, Danvers, MA, USA) and a rabbit monoclonal anti-collagen IV antibody (ab6586) (Abcam, Cambridge, UK). Color-coded high-molecular-weight (43-315 kDa) prestained protein markers (#12949) and a rabbit monoclonal anti-β-actin (13E5) antibody (#4970) were purchased from Cell Signaling Technology (Danvers, MA, USA). Goat anti-rabbit IgG-HRP (sc-2004) was purchased from Santa Cruz Biotechnology, Inc. (Delaware Ave., Santa Cruz, CA, USA). A Bicinchoninic Acid(BCA) Protein Assay Kit (P0012) and Sodium dodecyl sulfate polyacrylamide gel electropheresis (SDS-PAGE) Sample Loading Buffer (P0015) were purchased from Beyotime Institute of Biotechnology (Haimen, China). Real-Time-PCR was performed using a two-step RT-PCR FastQuant RT Kit (KR106) and SuperReal PreMix Plus (SYBR Green FP205) (Tiangen Biotech Company, Beijing, China). An E.Z.N.A.^®^ Total RNA Kit II (R6934-01) was purchased from OMEGA Bio-Tek (Norcross, GA, USA). The primers were designed using Primer Premier 5.0 (Premier, Palo Alto, CA, USA) and Oligo 6 software (Molecular Biology Insights, Cascade, CO, USA) and were synthesized by a biological engineering company (Shanghai, China).

### 2.2. Cell Lines and Cell Cultures

The murine colon adenocarcinoma cell line CT-26 was obtained from the Cell Bank of the Chinese Academy of Sciences (Shanghai, China). Cells were maintained in Roswell Park Memorial Institute (RPMI)-1640 medium supplemented with 15% fetal bovine serum and 1% penicillin-streptomycin. The cells were incubated in a humidified atmosphere containing 5% CO_2_ at 37 °C.

### 2.3. Animals and Treatment

Forty-eight female BALB/c mice (Shandong Lukang Pharmaceutical Co., Ltd., Qingdao, China; certificate of quality number: SCXK Lu 20130001) aged 6–8 weeks, with a mean weight of 18–22 g, were housed under specific pathogen-free (SPF) conditions. This study was approved by the medical ethics committee of Qingdao University Medical College. The mice were acclimated to the housing conditions (mean temperature of 24 ± 2 °C and mean humidity of 52% ± 8%) for 7 days. They were fed AIN-93M formula feed and filtered water. All mice were used in accordance with the institutional guidelines.

### 2.4. Liver Metastasis Animal Model

This study was approved by the medical ethics committee of Qingdao University Medical College (20141220, 2014). Tumor cells were infused into the portal system by injecting them into the splenic capsule and cutting the spleen. Tumor cell viability during the exponential growth phase was greater than 95%. A single-cell suspension was prepared in normal saline, and the density of injected cells was 1 × 10^6^/mL. The BALB/c mice were anesthetized via intraperitoneal injection of 1% sodium pentobarbital (80 mg/kg body weight) and fixed in the prone position. An approximately 1.5-cm incision was made in the abdomen between the dorsal central and left axillary midlines to expose the spleen. CT-26 tumor cells (1 × 10^6^ in 0.2 mL) were inoculated into the splenic tip using a 4-gauge needle to produce a visible pale wheal, followed by hemostasis for 5 min and wound closure. Blood vessels were ligated, and the spleen was removed. The abdominal wound was closed if no significant bleeding or extravasation was encountered.

### 2.5. Grouping and Treatment of Animals

Forty-eight SPF BALB/c mice were randomly distributed into the following four groups at one day after tumor cell inoculation: Experimental group 1: 80 mg/kg IP6; experimental group 2: 80 mg/kg Ins; experimental group 3: 80 mg/kg IP6 + Ins (IP6 40 mg/kg and Ins 40 mg/kg); and negative control group 4: Normal saline. The mice in each group received 0.2 mL of the treatment solution via gavage daily for 20 consecutive days. The IP6 + Ins solution was prepared at a 1:1 ratio (an *in vitro* experiment performed by our research group has shown that this ratio has the highest efficacy), and a volume of normal saline equal to that of the IP6 or Ins solution was administered to the control group. Notably, IP6 and Ins have molecular weights of 660.08 and 180, respectively.

### 2.6. Treatment of Experimental Animals

The mice were euthanized by cervical dislocation at 12 h after the final treatment. Then, they were inspected via gross dissection, and the sizes of the liver tumors were determined based on their weights. Liver specimens were fixed with 10% paraformaldehyde and cryosectioned coronally into 10-mm sections for routine hematoxylin and eosin staining. The liver metastasis inhibition rate was calculated as follows: liver metastasis inhibition rate (%) = [(average tumor weight of the control group − average tumor weight of the experimental group)/(average tumor weight of the control group)] × 100%.

### 2.7. Western Blot Analysis

Radioimmtmoprecipitation assay (RIPA) buffer containing Phenylmethanesulfonyl fluoride (PMSF) was used to extract total proteins from liver tumor tissues. The lysed tissues were centrifuged, and the supernatants were stored at −80 °C. Protein concentrations were measured using a BCA kit. Tissue lysates from each sample (50 µg) were subjected to SDS-PAGE using an 8% polyacrylamide gel with a 4% stacking gel, and the proteins were then transferred to polyvinylidene difluoride membranes. The membranes were blocked with 1% fat-free milk in Tris buffered saline -Tween(TBS-T) for 2 h and subsequently incubated with the following primary antibodies at 4 °C overnight: Anti-FN (diluted 1:1000), anti-LN (diluted 1:1000), anti-collagen IV (diluted 1:1000), and anti-β-actin (diluted 1:2000). Then, the membranes were incubated with a secondary antibody (diluted 1:5000) for 2 h. Chemiluminescent detection of bound antibodies was performed using an Enhanced chemiluminescence (ECL) PLUS system and a Molecular Imager ChemiDoc XRS System (Bio-Rad Laboratories, Hercules, CA, USA). Bands were identified and quantified using Image Lab Software (Version 2.0; Bio-Rad Laboratories, Berkeley, CA, USA).

### 2.8. Immunohistochemical Analysis

Livers were fixed with 10% paraformaldehyde. Paraffin-embedded tissues were used for immunohistochemical analysis. The liver tissues were selected for analysis while avoiding hemorrhage, necrotic, scar tissues, and the centers and edges of the specimens. A negative control group of tissues was established to prevent false positives. Incubations were performed at room temperature. Endogenous peroxidase activity was blocked with 3% hydrogen peroxide for 10 min. Then, the slides were washed, and nonspecific binding was blocked with 10% normal goat serum for 30 min. Next, the slides were incubated with the primary antibody diluted 1:200 at 4 °C overnight. The slides were subsequently washed with PBS and incubated with a biotinylated secondary antibody, an avidin-biotin horseradish peroxidase complex, and diaminobenzidine substrate according to the manufacturer’s specifications. The slides were counterstained with hematoxylin, dehydrated, and mounted. The stained slides were viewed and photographed using a Nikon Eclipse 80i microscope, and all photographs were captured under the same conditions. Positively stained cells were counted using Image-Pro Plus 6.0 software (Media Cybernetics, Georgia Avenue, Silver Spring, MD, USA). After performing light density correction, areas were selected for Integrated optical density (IOD) measurements. The selected areas were converted to grayscale images and then quantified. To reduce errors, images of the sections were adjusted to fill the entire field of view, with no large blank areas. The IOD values were used to represent the differences in staining among the samples.

### 2.9. Real-Time PCR

Total RNA was isolated from harvested (liver tumor) tissues using an E.Z.N.A.^®^ Total RNA Kit II (Norcross, GA, USA). mRNA levels were determined using an IMPLEN Nanophotometer P-330 nucleic acid detector (Munich, Germany). Total RNA (2 µg) was reverse transcribed using a Primer Script RT Reagent Kit and a Bio-Rad MyCycler PCR instrument (Hercules, CA, USA). Table 1 shows the sequences of the sense and antisense primers used and the resulting PCR product sizes. PCR was performed in a 20-µL reaction volume using an Eppendorf Mastercycler EP Gradient System (Eppendorf Hamburg, Germany) according to the manufacturer’s instructions. In this experiment, a two-step PCR cycling protocol was used, with a denaturation step at 95 °C and a combined annealing/extension step at 60 °C; this protocol is useful for primers with a Tm of well below 60 °C. The following cycling conditions were used: for collagen IV and LN: 50 cycles of 95 °C for 15 min, 95 °C for 10 s, and 60 °C for 25 s; for FN: 45 cycles of 95 °C for 15 min, 95 °C for 10 s, and 60 °C for 25 s; for TGF-β, integrin-β1, and MMP-9: 50 cycles of 95 °C for 15 min, 95 °C for 10 s, and 60 °C for 25 s; and for VEGF and bFGF: 55 cycles of 95 °C for 15 min, 95 °C for 10 s, and 60 °C for 25 s. The comparative Ct formula 2^−ΔΔ*C*t^ was used to calculate relative gene expression levels.

### 2.10. Statistical Analysis

All values are expressed as the mean ± standard deviation. Differences between groups were assessed by one-way analysis of variance. *p* < 0.05 was considered statistically significant. All statistical analyses were performed using SPSS software (version 17.0; IBM Corporation, NY, USA). Each experiment was repeated at least three times.

## 3. Results

### 3.1. Occurrence of Liver Metastasis in CRC in BALB/c Mice

**Figure 1:**
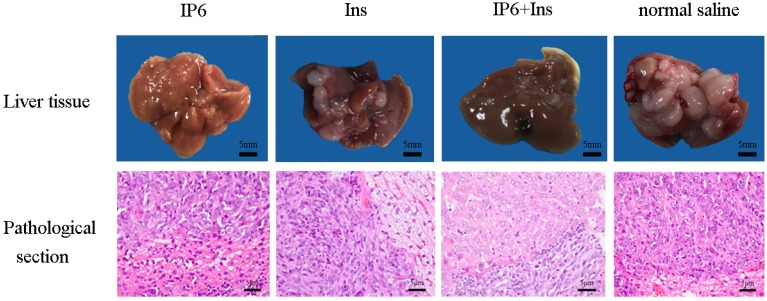
Upper panel, representative macroscopic appearances of mouse livers; nodules of different sizes were distributed on the liver surfaces. Lower panel, representative sections stained with hematoxylin and eosin, showing the histopathology of the mouse livers in the different groups. The magnification is 400×.

Mouse livers were dissected at the end of the experiment, and different-sized nodules were found to be distributed on their surfaces (Figure 1). Pathological biopsy confirmed that the liver nodules were poorly differentiated adenocarcinomas. In the control group, the tumor volume was large and covered the entire liver; in the Ins group, the tumor volume and distribution were reduced; in the IP6 group, the tumor volume was small, but the tumor distribution was broad; and in the IP6 + Ins group, the tumor volume was the smallest, and the tumors were concentrated in liver blood vessels. Pathological sections revealed that tumor cells were loosely arranged, with various shapes and sizes, hyperchromatic nuclei and nuclear fission, and they displayed increased nucleocytoplasmic ratios. In the IP6 group and Ins group, intense nuclear staining was detected. In contrast, in the IP6 + Ins group, weak nuclear staining was observed. In the control group, the nuclei appeared hyperchromatic, and the nucleocytoplasmic ratio was increased. The pathological sections of the poorly differentiated liver adenocarcinomas demonstrated the success of the mouse model, and mice lacking liver metastasis due to surgical failure or other reasons were excluded from the experiment. Table 2 shows the number of mice used, the tumor weights and the tumor inhibition rates. Compared with the control group, the IP6, Ins, and IP6 + Ins groups exhibited significantly reduced tumor weights, and the greatest effect was observed in the IP6 + Ins group. Relative to the control group, the tumor inhibition rates of the IP6, Ins, and IP6 + Ins groups were 53.52%, 52.15%, and 72.59%, respectively. Statistical analysis revealed that administration of IP6 or Ins significantly reduced the development and metastatic progression of CRC to the liver in the BALB/c mice and that the IP6 + Ins treatment had significantly greater effects than the other treatments.

### 3.2. Protein Expression of Collagen IV, LN and FN in Liver Tissue

**Figure 2:**
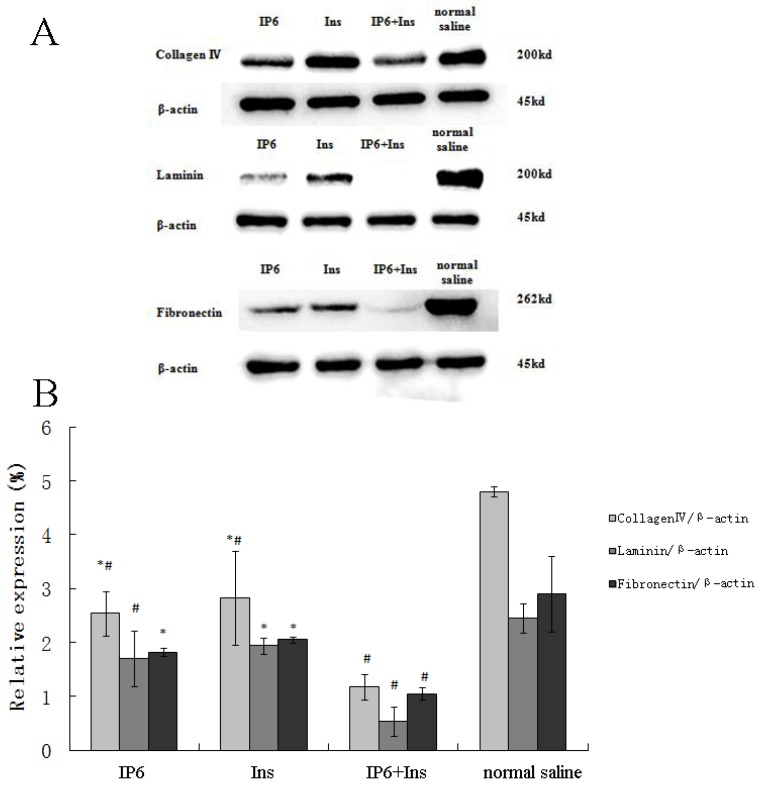
Western blot analysis of the effects of IP6, Ins, IP6 + Ins and normal saline on the levels of collagen IV, Lamininand Fibronectin. IP6 or Ins treatment decreased the protein expression of collagen IV, LN and FN, and the combined IP6 + Ins treatment resulted in significantly greater effects compared with treatment with either compound alone. The samples were probed with antibodies against p-collagen IV, p-LN, and p-FN. The Western blot membranes were stripped and reprobed for β-actin as an internal control to confirm equal loading. (**A**) representative blots from one of three separate experiments; (**B**) relative band intensities based on densitometry. The results are expressed as the mean ± standard deviation from three independent experiments. * *p* < 0.05 compared to the IP6 + Ins group; ^#^ *p* < 0.05 compared to the normal saline group.

To determine whether treatment with IP6, Ins or their combination alters the levels of the extracellular matrix (ECM) proteins collagen IV, LN and FN in the tumor metastasis microenvironment, Western blotting was performed to measure the expression of these proteins. Figure 2A shows the Western blot results from the IP6, Ins, IP6 + Ins and normal saline groups for these proteins. As shown in Figure 2B, collagen IV expression was significantly reduced from 4.79-fold to 2.54-, 2.83- and 1.17-fold in the IP6, Ins and IP6+Ins groups, respectively, relative to the control level. In addition, LN expression was significantly reduced from 2.44-fold to 1.69-, 1.94- and 0.52-fold and FN expression was significantly reduced from 2.90-fold to 1.82-, 2.05- and 1.05-fold in the IP6, Ins and IP6 + Ins groups, respectively, relative to the control level. Our analyses revealed that the treatment of BALB/c mice with IP6, Ins or their combination reduced the levels of ECM proteins (collagen IV, LN and FN) compared with their control levels and that the IP6 + Ins treatment significantly reduced the levels of these proteins (*p* < 0.01) compared with their levels following treatment with either compound alone.

### 3.3. Immunohistochemical Analyses of Integrin-β1, MMP-9, VEGF, bFGF, and TGF-β Protein Expression in Liver Tissue

**Figure 3:**
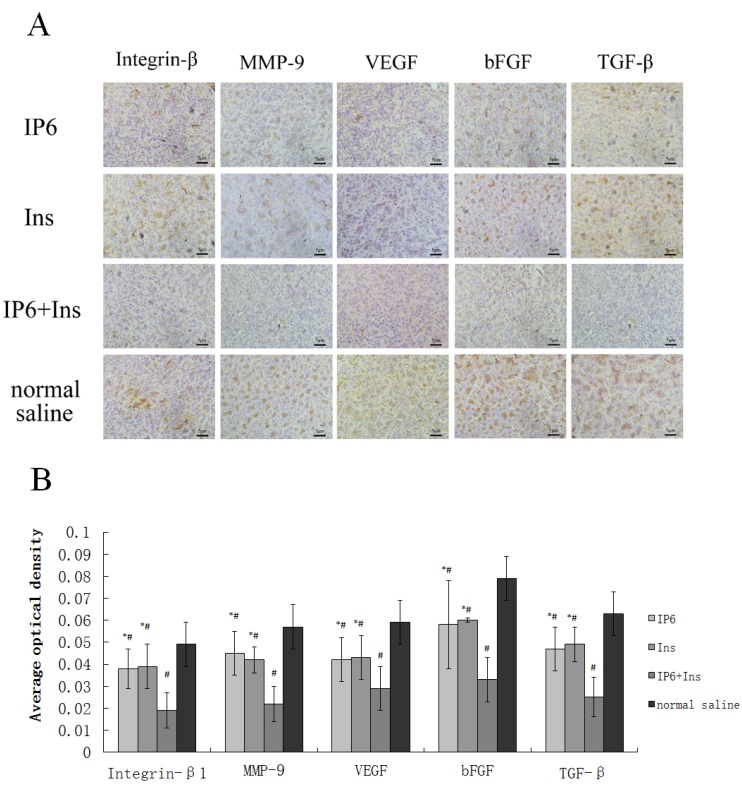
(**A**) Immunochemical analysis of vascular endothelial growth factor (VEGF), basic fibroblast growth factor (bFGF), transforming growth factor beta (TGF-β), integrin-β1, and matrix metalloproteinase (MMP)-9 protein expression in mouse liver tumor tissues; the expression of integrin-β1, MMP-9, VEGF, bFGF, and TGF-β was primarily localized to the cytoplasm. Positive staining appeared as brownish-yellow or brown granules under light microscopy; (**B**) the average optical densities of integrin-β1, MMP-9, VEGF, bFGF, and TGF-β protein staining revealed that the expression of these proteins was reduced in the IP6 group and Ins group compared to the control group; moreover, the combined application of IP6 and Ins further significantly reduced their expression. The results are expressed as the mean ± standard deviation from three independent experiments. * *p* < 0.05 compared to the IP6 + Ins group; ^#^ *p* < 0.05 compared to the normal saline group.

Qualitative and quantitative analyses of liver tissues were performed to determine whether treatment with IP6, Ins or their combination prevented metastasis of CRC to the liver. The protein expression of integrin-β1, MMP-9, VEGF, bFGF, and TGF-β was determined by immunohistochemical quantitation. As shown in Figure 3A, the expression of integrin-β1, MMP-9, VEGF, bFGF, and TGF-β was primarily localized in the cytoplasm. Positive staining appeared as brownish-yellow or brown granules under light microscopy. Immunostaining intensities were quantified using Image-Pro Plus software (Media Cybernetics, CA, USA) for computer-assisted image processing. Figure 3B shows the effects of the IP6, Ins, IP6 + Ins or normal saline treatment on the protein expression of integrin-β1, MMP-9, VEGF, bFGF, and TGF-β. Based on the average optical densities, the expression levels of these proteins were decreased in the liver tissues following the administration of IP6 or Ins. Moreover, their expression levels were significantly reduced in the mice in the control group compared with those in the IP6 + Ins group (integrin-β1: 60% reduction, *p* < 0.001; MMP-9: 60.4% reduction, *p* < 0.001; VEGF: 51.4% reduction, *p* < 0.001; bFGF: 57.6% reduction, *p* < 0.001; and TGF-β: 59.6% reduction, *p* < 0.001).

The IP6 and Ins groups also exhibited decreased expression of the following proteins compared with the control group: Integrin-β1: NS *vs.* IP6, 0.0497 ± 0.01003 *vs.* 0.0385 ± 0.00979, *p* = 0.028; NS *vs.* Ins, 0.0497 ± 0.01003 *vs.* 0.0391 ± 0.0148, *p* = 0.041; MMP-9: NS *vs.* IP6, 0.0566 ± 0.01068 *vs.* 0.0458 ± 0.01038, *p* = 0.01; NS *vs.* Ins, 0.0566 ± 0.01068 *vs.* 0.0424 ± 0.00660, *p* = 0.001; VEGF: NS *vs.* IP6, 0.0592 ± 0.01081 *vs.* 0.0423 ± 0.0108, *p* = 0.001; NS *vs.* Ins, 0.0592 ± 0.01081 *vs.* 0.0431 ± 0.01154, *p* = 0.003; bFGF: NS *vs.* IP6, 0.0791 ± 0.01146 *vs.* 0.0582 ± 0.01507, *p* < 0.001; NS *vs.* Ins, 0.0791 ± 0.01146 *vs.* 0.0601 ± 0.0094, *p* < 0.001; and TGF-β: NS *vs.* IP6, 0.06304 ± 0.06304 *vs.* 0.04714 ± 0.012741, *p* = 0.004; NS *vs*. Ins, 0.06304 ± 0.06304 *vs.* 0.04868 ± 0.04868, *p* = 0.01. Taken together, our results suggest that treatment with IP6 and Ins alone, and particularly treatment with their combination, inhibit tumor angiogenesis, invasion, and metastasis in a liver metastasis model of CRC in BALB/c mice.

### 3.4. Real-Time PCR Analyses of Collagen IV, LN, FN, Integrin-β1, MMP-9, VEGF, bFGF, and TGF-β mRNA Expression in Liver Tissue

**Figure 4:**
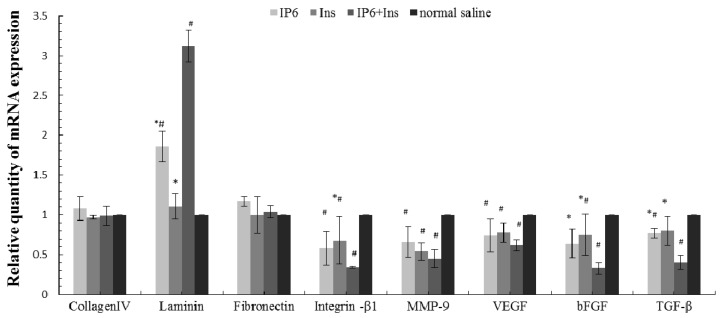
Real-time PCR analysis of the inhibitory effects of IP6, Ins, IP6 + Ins and normal saline on the mRNA expression of collagen IV, LN, FN, integrin-β1, MMP-9, VEGF, bFGF, and TGF-β. β-actin was used as a loading control. The collagen IV and FN mRNA levels did not significantly differ among the four groups. However, the LN mRNA level was higher in the experimental groups than in the control group. The IP6 or Ins treatment decreased the mRNA expression of integrin-β1, MMP-9, VEGF, bFGF, and TGF-β compared with the control treatment. The IP6 + Ins treatment also significantly reduced mRNA expression. These experiments were repeated in triplicate. * *p* < 0.05 compared to the IP6 + Ins group; ^#^ *p* < 0.05 compared to the normal saline group.

The mRNA expression levels of collagen IV, LN, FN, integrin-β1, MMP-9, VEGF, bFGF, and TGF-β were measured by real-time PCR to further characterize the mechanism by which CRC metastasizes to the liver and to determine whether IP6, Ins or their combination prevents the metastasis of CRC to the liver. The relative gene expression levels were calculated using the comparative Ct formula 2^−ΔΔ*C*t^. Figure 4 shows the effects of IP6, Ins, and IP6 + Ins compared with those of normal saline on expression of the target genes, specifically collagen IV, LN, FN, integrin-β1, MMP-9, VEGF, bFGF, and TGF-β. These experiments were repeated in triplicate. The collagen IV and FN mRNA levels did not significantly differ among the four groups. However, the LN mRNA level was higher in the experimental groups than in the control group, and this difference was significant among all groups except for the inositol and control groups (*p* < 0.05). IP6 or Ins treatment decreased the mRNA expression of integrin-β1, MMP-9, VEGF, bFGF, and TGF-β compared with the control treatment. IP6 + Ins treatment also resulted in significantly reduced mRNA expression (*p* < 0.05).

## 4. Discussion

Despite improvements in comprehensive therapies for CRC, clinicians treating patients with liver metastasis face challenges due to the limited efficacies of these therapies. To date, treatment with all available drugs in combination or in sequence until the occurrence of progression or unacceptable toxicity has been the standard therapy for metastatic colorectal cancer (mCRC) [19]. The aim of maintenance therapy is administration of an appropriate, minimally toxic regimen that does not compromise treatment efficacy or the quality of life of the patient [20]. The low toxicity and high safety of IP6 and Ins are advantageous in this field. In this study, we successfully established a liver metastasis model of CRC in BALB/c mice via infusion of CT-26 cells into the hepatic portal vein system by splenic capsule injection and spleen incision [21]. The tumor cells reached the liver sinus via blood circulation, adhered to sinus endothelial cells, and degraded the ECM to penetrate into the liver parenchyma and induce tumor angiogenesis. Our results demonstrated that IP6 and Ins inhibited the development of CRC metastasis to the murine liver by altering expression of the ECM proteins collagen IV, FN and LN, the adhesion factor receptor integrin-β1, the proteolytic enzyme MMP-9, and the angiogenic factors VEGF, bFGF, and TGF-β in the tumor metastasis microenvironment. The effects of the combined application of IP6 and Ins were greater than those of treatment with either IP6 or Ins alone.

Our team has been investigating the anti-tumor effects of IP6 for many years. Through *in vivo* and *in vitro* experiments, we have demonstrated that the anticancer activity of IP6 involves antioxidant activity [22]. Using human colon cancer HT-29 cells and hepatocellular carcinoma HepG2 cells, we have found that IP6 intervention modulates cellular signal transduction [23,24]. IP6 has an inhibitory effect on HT-29 cells through regulation of the PI3K/Akt pathway; in particular, it has an inhibitory effect on the expression of PI3K, Akt and pAkt, whereas it promotes expression of its downstream signaling target, caspase-9. Studies have also demonstrated that IP6 is involved in regulation of the cell cycle [25,26], inhibition of proliferation [27,28,29], induction of differentiation [30] and promotion of apoptosis [31,32]. These effects are likely attributed to lower-phosphate inositol phosphates (such as IP1-5). The application of Ins, the molecular backbone of IP6, increases the quantity of its lower phosphorylated forms, which synergistically interact with IP6 to exert stronger anticancer effects. IP6 has been shown to inhibit the growth of transplanted HT-29 cell-based tumors in nude mice [33]. In this study, we first investigated the effects of IP6 and inositol on metastatic tumors.

Metastasis of CRC to the liver is a dynamic process, and successful invasion and metastasis require alterations in the corresponding molecular mechanisms. The process of tumor invasion and metastasis involves cancer cells, the adjacent matrix, growth factors, cytokines, the ECM, enzymes, and liver cell components, all of which are needed to support metastasis in the tumor microenvironment. The three main components of the cell-ECM interaction are collagen IV, FN and LN, while transmembrane cell surface receptors termed integrins serve as links that transduce signals from the outside to the inside of the cell. Tumor cells continuously exchange material with the ECM via integrins, and these events affect the proliferation, apoptosis, migration and gene expression of cancer cells. Conversely, cancer cells and stromal cells secrete a variety of proteins and enzymes that induce ECM synthesis or promote its degradation. MMPs and tissue inhibitors of metalloproteinases (TIMPs) constitute the main protein hydrolysis enzymes in the ECM, and imbalances in their dynamic equilibrium affect adhesion between tumor cells and provide the basis for tumor angiogenesis [34]. Tumor growth and metastasis require the formation of new blood vessels, and tumor cells secrete the angiogenic growth factors VEGF, bFGF, and TGF-β, which also promote tumor vascular endothelial cell proliferation.

The ECM is an important component of the microenvironment because it provides structural support for tumor cells and acts as a signaling factor to regulate the functions of tumor cells and vascular endothelial cells [35]. Collagen IV interacts with different cell surface receptors involved in various intracellular signaling pathways to promote tumor cell proliferation and migration as well as tumor angiogenesis [36]. FN, a potential diagnostic marker of liver cancer invasion and metastasis, participates in forming the cytoskeleton and in determining cell morphology [37]. LN and its related proteins play an important role in maintaining the normal function of the basement membrane, which is involved in cell growth, differentiation, migration, and adsorption [38]. Our study demonstrated that IP6 and Ins reduced the protein expression of collagen IV, FN and LN, and that their combination had even greater effects. The mRNA expression of collagen IV, FN and LN was not reduced by treatment with IP6, Ins, or IP6 + Ins. Naba Alexandra *et al.* have compared ECM assessment methods and have demonstrated that the ECM protein levels are more appropriate indicators of tumor properties than the ECM mRNA levels because ECM proteins are the functional molecules in the tumor microenvironment; in addition, changes that occur at the protein level are not reflected at the mRNA level because of post-transcriptional processes (e.g., translation and protein stability) [36]. However, in our study, the IP6 and IP6 + Ins treatments caused up-regulation of LN mRNA expression. Spenle *et al.* have shown that high laminin α1 or laminin α5 expression decreases susceptibility to experimental dextran sodium sulfate (DSS)-induced colon inflammation, as assessed via histological scoring, and decreases the levels of pro-inflammatory cytokines [39]. The IP6 and IP6 + Ins treatments performed in our study increased the mRNA expression of LN and may have decreased the levels of pro-inflammatory cytokines, affecting tissue repair; however, the protein level of LN depends not only on its synthesis but also on its degradation, as well as other factors. Other studies have found that certain tumor-derived ECM proteins exhibit characteristics of high tumor metastasis and play important roles in this process [40]. The IP6-induced decreases in ECM protein levels may be due to its inhibition of tumor cell proliferation and promotion of tumor cell apoptosis. In addition, IP6 enhances the differentiation of malignant cells into more mature cells, often resulting in their reversion to a normal phenotype [41]. This effect leads to a reduction in cell–ECM interactions and the adhesion of tumor cells, resulting in a lack of structural support for tumor cell migration. Consistent with our findings, an interventional experiment using MDA-MB-231 breast cancer cells, which exhibit a high invasive ability *in vitro*, has demonstrated that IP6 reduces the ability of cancer cells to adhere to collagen IV, FN, and LN and suppresses the capacity of MDA-MB-231 cells to invade and restructure the basement membrane [42].

Integrin-β1 is a member of a group of receptors that bind to Arg-Gly-Asp (RGD) sequences in the plasma membrane. It is an important cell adhesion molecule that is involved in many physiological functions and pathological changes. It is highly expressed in liver metastatic CRC tumor cells [43]. The immunohistochemistry and RT-PCR results obtained in this study demonstrated that the IP6 and Ins treatments decreased the protein and mRNA expression levels of integrin-β1 and that the combined IP6 + Ins treatment exerted the strongest effects. ECM proteins and intracellular cytoskeletal proteins are attached via integrin-β1 and form focal adhesion molecules, which activate the PI3K signaling pathway to regulate cell apoptosis and proliferation [44]. IP6 may exert its anti-metastatic activity by altering integrin expression and the activities of downstream signaling pathways. Our previous studies have demonstrated that IP6 inhibits both PI3K and AP-1 activation [23]. Akt, a downstream signal of PI3K that is responsible for cell survival, was also inactivated by IP6 [45,46]. Similarly, *in vitro* experiments have revealed that IP6 reduces integrin-β1 expression and cell adhesion and migration by inhibiting integrin-mediated cell signaling pathways via focal adhesion kinase (FAK) phosphorylation [46].

MMP-9 is a type IV collagenase, and its high expression is closely correlated with malignant tumor invasion, metastasis and vascular formation via ECM and basement membrane degradation and damage near the tumor surface. This damage causes the release of many growth-promoting factors and tumor cells into surrounding tissues, thereby promoting tumor invasion and metastasis [47,48]. Our results suggest that IP6 and Ins exert moderate anticancer effects by inhibiting MMP-9 expression and that the combined IP6 + Ins treatment has the greatest effects. The anticancer activity of IP6 may also be related to its mineral binding ability [49]. MMPs are zinc-dependent endopeptidases, and one possible mechanism by which IP6 inhibits MMP expression involves its high affinity for divalent minerals, which could prevent the activation of Zn^2+^-dependent MMPs [50]. Active MMPs target ECM ligands (e.g., LN, collagen, and FN) for degradation, which results in cancer cell detachment from the ECM and ECM remodeling [16]. MMP expression in tumor tissues is regulated by growth factors and cytokines secreted by tumor cells, such as the tumor angiogenic factor bFGF, or by the epithelial-mesenchymal transition (EMT), which increases MMP activity [51]. IP6 has also been shown to exhibit anti-angiogenic activity and may thus indirectly inhibit MMP-9 activity [52].

Tumor angiogenesis is particularly important for the metastasis of lesions. It is mediated by the release of angiogenic factors, including VEGF, bFGF and TGF-β, all of which promote degradation of the ECM and basement membrane, thereby promoting local tumor invasion and distant metastasis. VEGF, the most specific angiogenic factor, is secreted by tumor cells and promotes endothelial cell proliferation and the remodeling of new vessels [52]. A high level of VEGF is a significant predictor of metachronous liver metastasis and hepatic recurrence following the synchronous resection of liver metastases [53]. VEGF and bFGF, both of which bind to fibrinogen and fibrin, perform distinct and complementary functions in angiogenesis [54]. bFGF, the most potent tumor vascular growth factor, promotes the proliferation of vascular endothelial cells, which induce the migration of capillary endothelial cells to the three-dimensional collagen matrix to form a shallow, capillary-like structure. The abnormal expression of bFGF promotes cell proliferation, malignant transformation and tumor formation [55]. TGF-β, a cytokine with complex biological functions, plays roles in both VEGF activation and microvascular remodeling. Elevated expression of TGF-β in CRC is positively associated with subsequent liver metastasis [56]. After liver metastasis of CRC occurs, the metastatic liver tumor continues to secrete large amounts of TGF-β via autocrine and paracrine pathways. In turn, TGF-β can be induced via the stimulation of angiogenesis to induce cell spreading and suppress immune functions, thereby promoting the further development of liver metastases from CRC [57].

The results of our study have demonstrated that IP6 and Ins modulate angiogenesis and decrease the protein and mRNA levels of VEGF, bFGF and TGF-β, although the effect of each agent alone is relatively moderate. The combined application of IP6 and Ins had the most significant anticancer effects. VEGF, bFGF and TGF-β promote endothelial cell proliferation and the remodeling of new vessels. Ivana Vucenik *et al.* have reported that angiogenesis depends on interactions between vascular endothelial cells and tumor cells; because IP6 affects both cell types, it may disrupt the formation of new vessels by inhibiting secretion of the angiogenic factors VEGF and bFGF [52]. VEGF and bFGF bind to their receptors on cell membranes to perform their biological functions, including roles in the Ras/Raf/MAPK [58], PI3K/Akt [59] and PI3K/PKC pathways [60], to promote vascular endothelial cell growth and increase vascular permeability. Marks *et al.* have revealed that TGF-β is highly expressed in CRC tissues induced by azoxymethane (AOM) and that IP6 intervention significantly reduces TGF-β expression [61]. TGF-β promotes the development of tumor angiogenesis, which is related to the TGF-β/Smad signal transduction pathway, as well as alternative pathways (e.g., the MAPK, PI3K/AKT, and NF-κB pathways) [62,63,64]. IP6 blocks the PI3K/Akt, AP-1 [23,65,66], PI3K/PKC [67] and MAPK [68] signal transduction pathways to regulate the cell cycle, thereby blocking uncontrolled cell division and forcing malignant cells to either differentiate or enter apoptosis. These effects can directly or indirectly inhibit expression of the vascular growth factors VEGF, bFGF and TGF-β.

## 5. Conclusions

To our knowledge, this is the first study to evaluate the anti-metastatic effects of IP6, Ins and their combination *in vivo* in a mouse liver metastasis model. Additionally, we first attempted intragastric administration because IP6 is more easily degraded to lower-phosphate inositol phosphates by phytases in the gastrointestinal tract, and these compounds also exhibit effective anti-cancer effects. We used the splenic capsule injection method to establish a model of CRC metastasis to the liver, as this method only simulates tumor cell migration via blood circulation and the metastasis of tumor cells. We will investigate the metastasis of primary cancers in future studies. IP6 and Ins are present in abundance in regular diets, and they are safely and efficiently absorbed from the gastrointestinal tract. The combined application of IP6 and Ins may have broad impacts on the prevention and treatment of cancer.

## Figures and Tables

**Table 1:** Real-Time PCR primer sequences used and sizes of products obtained.

Gene	Primer Sequence	Product Size (bp)
β-actin	F 5′-TTCCTTCTTGGGTATGGAAT-3′	174
	R 5′-GAGCAATGATCTTGATCTTC-3′	
Collagen IV	F 5′-CCTCCAGGTTTCCCTACTCC-3′	123
	R 5′-GGCTCCATCTCTTCCACTTG-3′	
Laminin	F 5′-AGTACCAGGAGGAAGCAGCA-3′	126
	R 5′-TCTAAGCATCGCAAGGGAGT-3′	
Fibronectin	F 5′-ACCACCCAGAACTACGATGC-3′	124
	R 5′-CATGCTGCTTATCCCACTGA-3′	
Integrin-β1	F 5′-CAGTGAATGGCAACAATGAAG-3′	133
	R 5′-ATCAGCAGCAAGGCAAGG-3′	
Matrix metalloproteinase 9	F 5′-TCTACTGGGCGTTAGGGACA-3′	112
	R 5′-TCGGGAGAGAGAGGAGTCTG-3	
VEGF	F 5′-CCCTTCGTCCTCTCCTTACC-3′	118
	R 5′-AAGCCACTCACACACACAGC-3′	
bFGF	F 5′-CGGTCACGGAAATACTCC-3′	120
	R 5′-GCTCTTAGCAGACATTGGAAG-3′	
TGF-β	F 5′-ATTCCTGGCGTTACCTTGG-3′	120
	R 5′-AGCCCTGTATTCCGTCTCCT-3′	

**Table 2:** The occurrence of tumors in experimental animals ($\bar{x}$ ± S).

Group	Number	Tumor Weight (g)	Inhibition Rate (%)
IP6	9	0.541 ± 0.349 *^,^^#^	53.52
Ins	9	0.557 ± 0.421 *^,#^	52.15
IP6 + Ins	9	0.319 ± 0.117 ^#^	72.59
Normal saline	11	1.164 ± 0.558	-

* *p* < 0.05 compared to the IP6 + Ins group; ^#^ *p* < 0.05 compared to the normal saline group.
